# A randomized porcine study of hemorrhagic shock comparing end-tidal carbon dioxide targeted and proximal systolic blood pressure targeted partial resuscitative endovascular balloon occlusion of the aorta in the mitigation of metabolic injury

**DOI:** 10.1186/s40635-023-00502-w

**Published:** 2023-04-10

**Authors:** Anna Stene Hurtsén, David T. McGreevy, Christina Karlsson, Claes G. Frostell, Tal M. Hörer, Kristofer F. Nilsson

**Affiliations:** 1 Department of Cardiothoracic and Vascular Surgery, Faculty of Medicine and Health, Örebro University, Örebro, Sweden; 2 Centre for Clinical Research and Education, County Council of Värmland, Karlstad, Sweden; 3 School of Medical Sciences, Örebro University, Örebro, Sweden; 4 School of Health Sciences, Örebro University, Örebro, Sweden; 5 Anesthesiology and Intensive Care, Department of Clinical Sciences, Karolinska Institute at Danderyd Hospital, Stockholm, Sweden; 6 Department of Surgery, Faculty of Medicine and Health, Örebro University, Örebro, Sweden

**Keywords:** Chock, hemorrhagic, Balloon occlusion, Ischemia–reperfusion injury, Carbon dioxide, Metabolism

## Abstract

**Background:**

The definition of partial resuscitative endovascular balloon occlusion of the aorta (pREBOA) is not yet determined and clinical markers of the degree of occlusion, metabolic effects and end-organ injury that are clinically monitored in real time are lacking. The aim of the study was to test the hypothesis that end-tidal carbon dioxide (ETCO_2_) targeted pREBOA causes less metabolic disturbance compared to proximal systolic blood pressure (SBP) targeted pREBOA in a porcine model of hemorrhagic shock.

**Materials and methods:**

Twenty anesthetized pigs (26–35 kg) were randomized to 45 min of either ETCO_2_ targeted pREBOA (pREBOA_ETCO2_, ETCO_2_ 90–110% of values before start of occlusion, *n* = 10) or proximal SBP targeted pREBOA (pREBOA_SBP_, SBP 80–100 mmHg, *n* = 10), during controlled grade IV hemorrhagic shock. Autotransfusion and reperfusion over 3 h followed. Hemodynamic and respiratory parameters, blood samples and jejunal specimens were analyzed.

**Results:**

ETCO_2_ was significantly higher in the pREBOA_ETCO2_ group during the occlusion compared to the pREBOA_SBP_ group, whereas SBP, femoral arterial mean pressure and abdominal aortic blood flow were similar. During reperfusion, arterial and mesenteric lactate, plasma creatinine and plasma troponin concentrations were higher in the pREBOA_SBP_ group.

**Conclusions:**

In a porcine model of hemorrhagic shock, ETCO_2_ targeted pREBOA caused less metabolic disturbance and end-organ damage compared to proximal SBP targeted pREBOA, with no disadvantageous hemodynamic impact. End-tidal CO_2_ should be investigated in clinical studies as a complementary clinical tool for mitigating ischemic–reperfusion injury when using pREBOA.

## Introduction

Resuscitative endovascular balloon occlusion of the aorta (REBOA) is an emerging endovascular tool in the management of life-threatening non-compressible hemorrhage [1, 2]. The aortic balloon technique originated during the Korean war [3], had a modern upsurge in the endovascular management of ruptured abdominal aortic aneurysm and is now recognized as a tool in the Endovascular Resuscitation and Trauma Management (EVTM) concept, including indications other than trauma [1, 4–9]. The aim is to decrease an ongoing bleeding but preserve cerebral and myocardial perfusion until definite hemostasis is achievable [1, 2, 8, 10, 11]. However, the supra-physiologic blood pressure proximal to the occlusion can lead to cardiac failure and worsening of traumatic brain injury [12–14]. Moreover, distal ischemia and subsequent reperfusion injury upon deflation of the balloon is challenging and highly dependent on occlusion time [1, 7, 13–17]. To overcome these complications, new techniques have been developed. Partial REBOA (pREBOA) allows a permissible blood flow over the balloon and thereby restrictively perfuse distal organs [18]. By permitting some circulation distal of the occlusion, anaerobic metabolism is restricted, thus limiting the accumulation of metabolites and subsequent metabolic acidosis, which occurs in total aortic occlusion. In addition, the ischemic burden on the kidneys and intestines is reduced compared to total occlusion [19, 20]. Partial REBOA may enable extended occlusion time and may diminish rebound hypotension upon deflation which occasionally leads to reinflation [19, 21]. The definition of “partial” is, however, not yet concluded and clinical markers of the degree of occlusion, metabolic disturbance and end-organ injury that can be easily monitored in real time are lacking. In research settings, balloon titration has been correlated with targets of proximal systolic blood pressure (SBP) alone, pressure gradient proximal to distal over the balloon, intra-balloon pressure and volume, Computer Tomography imaging or by specially designed devices to control distal blood flow [19, 20, 22–25]. None of these indices alone provide enough information about tissue perfusion and metabolic state at organ level [14, 19–21]. End-tidal carbon dioxide (ETCO_2_) has been suggested as an indicator in the use of Preboa, since it correlates to oxygen consumption [20, 26]. End-tidal CO_2_ estimates oxygen consumption, since delivered oxygen converts to carbon dioxide during cell metabolism [27]. End-tidal CO_2_ is used clinically and can be continuously measured in all intubated patients, and associates to cardiac output in critically ill trauma patients [28]. It could, therefore, be a useful noninvasive indicator of changes in systemic oxygen consumption and thereby a potential marker of metabolic disturbance and eventual end-organ injury caused by pREBOA. The aim of this study was to test the hypothesis that ETCO_2_ targeted pREBOA causes less metabolic damage (determined by arterial lactate concentrations at 1 h of reperfusion as primary outcome) and end-organ damage compared to proximal SBP targeted pREBOA in a porcine model of controlled hemorrhagic shock.

## Materials and methods

### Study design

An interventional study approved by the Regional Animal Ethics Committee (ID 1525-2019, Linköping Sweden March 7, 2019) was conducted at the animal research laboratory, Örebro University, Örebro, Sweden. All animal handling was in compliance with the directives of the European Parliament and the Council on the protection of animals used for scientific purposes and conduced according to the Replacement, Reduction and Refinement principles [29]. Twenty-nine Swedish country breed (Hampshire and English Yorkshire cross breed) pigs were obtained from a local farmer at 3–4 months, with a mean weight of 30 kg (26–35 kg) and a gender ratio of approximately 1:1. Each group included 10 animals based on an interim analysis of arterial lactate concentrations as primary outcome. An a priori exclusion criterium was that animals that died before conclusion of the experiment should be replaced with new animals. The study adhered to the ARRIVE guidelines as closely as feasible [30].

### Surgical preparation, anesthesia, and monitoring

The animals were premedicated with azaperone and anesthetized with tiletamine/zolazepam ± propofol as previously described as previously described [20, 31]. Anesthesia was maintained by Propofol 10 mg kg^−1^ h^−1^ (Fresenius Kabi, Uppsala Sweden) and Remifentanil 40 µg kg^−1^ h^−1^ (Actavis, Dublin, Irland) using infusion pumps (Alaris GP; Cardinal Health Care, Rolle, Switzerland). At baseline, volume-controlled ventilation (Airox™ Legendair™, Coviden, Hampshire, UK) was set to tidal volumes 10 ml kg^−1^ and the respiratory rate was adjusted to maintain ETCO_2_ at 5.0 ± 0.5%, except during the hemorrhage and intervention periods. Body temperature (measured through the pulmonary arterial catheter) was maintained at 39 ± 0.5 °C using heat blankets.

Arterial and venous accesses for hemodynamic measurements, blood sampling, controlled hemorrhage, endovascular intervention and fluid administration were obtained in accordance with a previous study [20]. In short, arterial access was gained through the right common carotid artery (5 Fr) and the femoral artery bilaterally (10 Fr in the right, 4 Fr in the left) and venous access was gained through the external jugular vein bilaterally (10 Fr). A pulmonary arterial catheter (7.5 Fr, CCOMBO, Edwards Lifesciences, Irvine, CA, USA) was inserted through the right venous sheath. A baby feeding catheter (Nutrisafe2, Vycon, Ecouen, France) was placed into a mesenteric vein for blood sampling. A flow probe (Vascular TTFM Probe 10 mm; Medistim ASA, Oslo, Norway) was placed over the aorta at diaphragm level through laparotomy and the urinary bladder was catheterized through a cystotomy (12 Fr, Foley; Bard Limited, Crawley, UK). The abdomen was closed and Heparin 5000 IE (LEO Pharma, Malmö, Sweden) was administered. An intervention-free hour was allowed for hemodynamic stabilization before the start of the experiment.

### Protocol and study groups

**Fig. 1:**
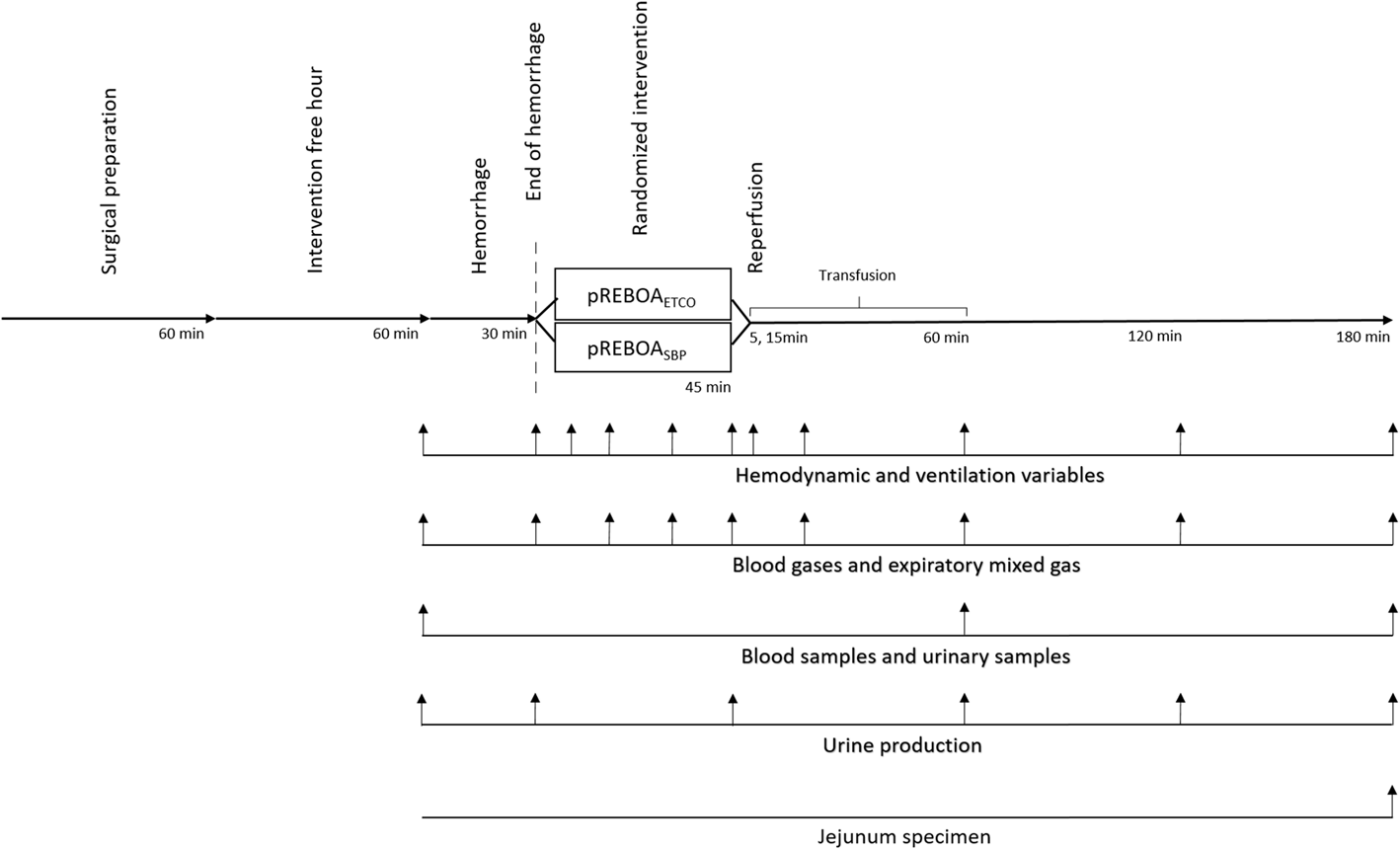
Experimental protocol of partial endovascular balloon occlusion of the aorta (pREBOA) targeted by end-tidal carbon dioxide (pREBOA_ETCO2_, *n* = 10) and proximal systolic blood pressure (pREBOA_SBP_, *n* = 10) in anesthetized, mechanically ventilated pigs. Each arrowhead indicates a measurement point

After stabilization, controlled hemorrhage was induced through the right femoral access (Fig. 1). Thirty percent of the calculated blood volume (66 ml kg^−1^) was eliminated over 15 min, additional blood was withdrawn over a further 15 min, aiming for a SBP of 55–60 mm Hg. The blood was collected in citrated bags (Fenwal™, Baxter, Illinois, USA) for later autologous transfusion. A REBOA catheter (Rescue Balloon; Tokai Ltd, Japan) was then inserted through the same access and advanced to the thoracic aorta (zone I) using landmarks; correct position was confirmed by blood pressure elevation and reduction of aortic blood flow. The subjects were block randomized by envelop system to either proximal SBP targeted (pREBOA_SBP_) or ETCO_2_ targeted inflation of the REBOA (pREBOA_ETCO2_). The SBP target was set at 80–100 mm Hg and the ETCO_2_ target at 90–110% of the reference value at the end of hemorrhagic. If necessary, the inflation volume was manually adjusted by 0.25 ml after every measurement point (i.e., every 15 min) to maintain the pre-set interval. After 45 min of occlusion, the balloon was gradually deflated over 5 min. Two pigs, one in each group, received Epinephrine 0.1 mg (Martindale Pharma, Romford, UK) when deflating the balloon to counteract abrupt hypotension. Fluid administration (5% glucose 1 ml kg^−1^ and Ringer’s Acetate 10 ml kg^−1^) was stopped during hemorrhage and occlusion and restarted at reperfusion in addition to autologous blood transfusion. One transfusion bag (500 ml) was transfused within 15 min, the remaining withdrawn blood was transfused over the next 45 min. Additional 20 ml glucose (30 mg ml^−1^, Fresenius Kabi, Uppsala, Sweden) was given if glucose levels dropped below 3.0 mmol L^−1^. If required, an extra blood gas was taken after 30 min for evaluation. Ventilation settings were fixed during hemorrhage and intervention. Respiratory frequency was adjusted after 1 h of reperfusion to achieve ETCO_2_ at 5.0 ± 0.5%. Hemodynamic and respiratory variables were monitored on an intensive care monitor (AS/3, Datex, Helsinki, Finland), collected by MP150/Acknowledge 3.9.1 system (BIOPAC systems; Goleta, CA, USA), and data were sampled according to the protocol (Fig. 1). Airtight bags were used to collect exhaled gas for analysis of the fractions of mixed exhaled gases in the intensive care monitor (AS/3, Datex, Helsinki, Finland). Arterial, mixed-venous and mesenteric blood gases, arterial and mesenteric blood samples, and urine samples were collected at the indicated timepoints (Fig. 1). After 3 h of reperfusion, a specimen from the jejunum was collected and preserved in 4% Formaldehyde solution (Solveco AB, Rosersberg, Sweden) for blinded evaluation by a pathologist using a six-grade system, where 0 represents normal tissue and 6 represents villi without epithelium (dead villi) and crypt destruction [6, 32, 33]. Euthanasia was performed with 20 ml potassium chloride (2 mmol ml^−1^, Braun, Danderyd, Sweden) after a bolus dose of Propofol (200 mg). Cardiac arrest was confirmed by SBP, electrocardiography and ETCO_2_. The primary outcome variable was arterial lactate concentrations at 1 h of reperfusion.

### Calculations

Oxygen consumption (VO_2_) was calculated as (inspired fraction of oxygen − fraction of mixed exhaled oxygen) × minute ventilation (MV). Carbon dioxide production (VCO_2_) was calculated as fraction of mixed exhaled carbon dioxide × MV. Oxygen delivery (DO_2_) was calculated as CO × arterial content of oxygen and oxygen extraction ratio (O_2_ER) was calculated as (arterial content of oxygen − mixed-venous content of oxygen)/arterial content of oxygen. Arterial and mixed-venous content of oxygen was calculated as Hb × oxygen saturation × 1.34 + pO_2_ × 0.23 using values from arterial and mixed-venous blood samples, respectively.

### Statistical analysis

Statistical analyses were not blinded. Normal distribution was examined using the Shapiro–Wilk test and non-normally distributed data were transformed by the logarithm (aspartate aminotransferase, troponin I and diuresis) and reanalyzed using the Shapiro–Wilk test. A linear mixed model with group, time and their interaction was used in an autoregressive model using IBM SPSS version 26 (SPSS Inc., Chicago, IL. USA). If the interaction between group and time was statistically significant, a Bonferroni-adjusted post-hoc multiple comparison analysis was performed. Mann–Whitney *U* test was used for statistical analysis of histologic samples. Statistical significance was considered as *p* < 0.05. Graphs were made in GraphPad prism version 8 (GraphPad Software Inc, San Diego, CA, USA). Data are presented as means with confidence intervals unless otherwise indicated.

## Results

Four animals died during hemorrhage before randomization and were excluded from the study. Further two animals died during reperfusion in the pREBOA_SBP_ group and one in the pREBOA_ETCO2_ group. In addition, one animal in the pREBOA_SBP_ group was excluded as an outlier (decided by the first and last authors due to deviation from the mean by more than two standard deviations in several variables) and one in the pREBOA_ETCO2_ group due to accidental air pulmonary embolism. All excluded animals were replaced according to the pre-determined protocol, resulting in 10 animals in each group completing the study and included in the statistical analysis.

### Hemorrhage

**Fig. 2:**
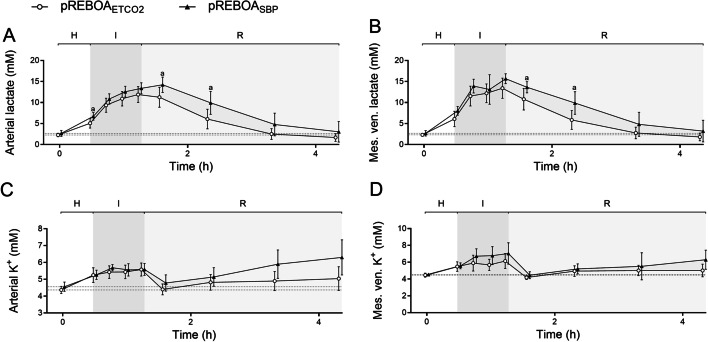
End-tidal carbon dioxide (ETCO_2_, **A**), systemic blood pressure (SBP, **B**), abdominal aortic blood flow (ABF, **C**) and femoral arterial mean pressure (FMP, **D**) in anesthetized, mechanically ventilated pigs undergoing hemorrhage (H), intervention (I) using either end-tidal carbon dioxide (ETCO_2_) targeted partial resuscitative endovascular balloon occlusion of the aorta (pREBOA, pREBOA_ETCO2_, *n* = 10) and proximal systolic blood pressure targeted pREBOA (pREBOA_SBP_, *n* = 10) and subsequent reperfusion (R). ^a^ Denotes statistical difference between the groups at a given timepoint. Data are means (95% confidence interval). The dotted line represents baseline

**Fig. 3:**
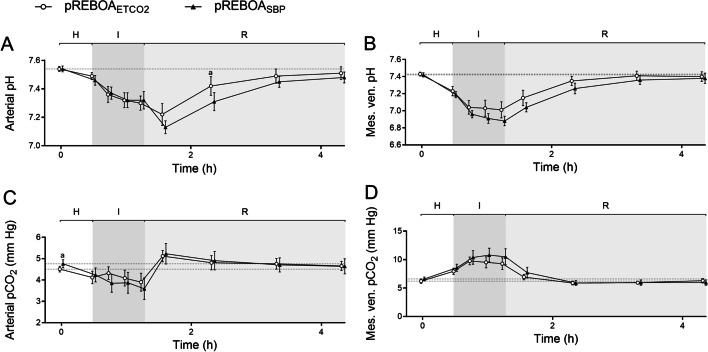
Arterial and mesenteric venous pH (**A**, **B**) and pCO_2_ (**C**, **D**) in anesthetized, mechanically ventilated pigs undergoing hemorrhage (H), intervention (I) using either end-tidal carbon dioxide (ETCO_2_) targeted partial resuscitative endovascular balloon occlusion of the aorta (pREBOA, pREBOA_ETCO2_, *n* = 10) and proximal systolic blood pressure targeted pREBOA (pREBOA_SBP_, *n* = 10) and subsequent reperfusion (R). ^a^ Denotes statistical difference between the groups at given timepoint. Data are means (95% confidence interval). The dotted line represents baseline

**Fig. 4:**
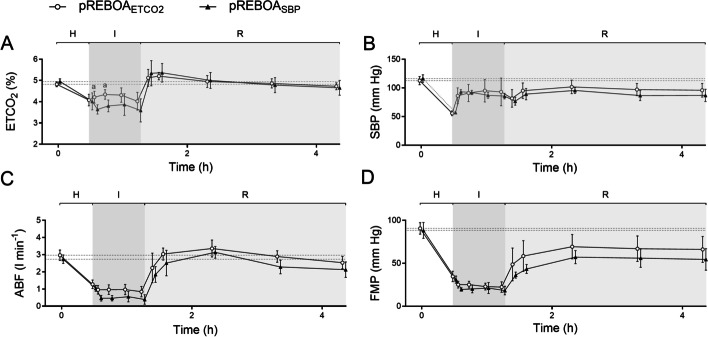
Arterial and mesenteric venous lactate (**A**, **B**) and potassium (K; **C**, **D**) concentrations in anesthetized, mechanically ventilated pigs undergoing hemorrhage (H), intervention (I) by end-tidal carbon dioxide (ETCO_2_) targeted partial resuscitative endovascular balloon occlusion of the aorta (pREBOA, pREBOA_ETCO2_, *n* = 10) and proximal systolic blood pressure targeted pREBOA (pREBOA_SBP_, *n* = 10) and subsequent reperfusion (R). ^a^ Denotes statistical difference between the groups at given timepoint. Data are means (95% confidence interval). The dotted line represents baseline

The groups were equivalent at baseline except for arterial pCO_2_ (Figs. 2, 3, 4, Tables 1, 2). Hemorrhage caused a hemodynamic shock state with systemic hypotension, SBP was 56 (CI 52–60) mm Hg in the pREBOA_ETCO2_ group and 57 (CI 55–59) mm Hg in the pREBOA_SBP_ group (Fig. 2, Table 1), which corresponded to a blood loss of 47% and 50% of total blood volume, respectively.

**Table 1:** Hemodynamics during partial resuscitative endovascular balloon occlusion of the aorta

	Baseline	Hemorrhage	15 min pREBOA	30 min pREBOA	45 min pREBOA	15 min reperfusion	60 min reperfusion	120 min reperfusion	180 min reperfusion
HR (beats min^−1^)									
pREBOA_ETCO2_	117 (101–132)	163 (137–189)	175 (145–204)	192 (153–220)	181 (158–204)	173 (156–191)	165 (148–183)	143 (125–162)	140 (116–165)
pREBOA_SBP_	127 (107–148)	206 (192–220)	198 (176–220)	211 (197–225)	199 (184–214)	160 (152–168)	169 (158–181)	161 (140–183)	156 (132–179)
MAP (mm Hg)									
pREBOA_ETCO2_	92 (85–100)	35 (30–40)	71 (58–84)	73 (57–89)	69 (51–87)	61 (46–75)	72 (60–85)	71 (59–82)	69 (58–81)
pREBOA_SBP_	94 (86–103)	34 (29–38)	66 (60–72)	58 (48–69)	62 (55–70)	44 (40–48)	59 (54–64)	59 (50–64)	58 (47–69)
CVP (mm Hg)									
pREBOA_ETCO2_	7 (6–8)	4 (3–5)	5 (4–6)	5 (4–6)	5 (4–6)	7 (6–8)	7 (6–8)	6 (6–7)	7 (6–8)
pREBOA_SBP_	9 (8–10)	5 (4–6)	7 (6–8)	7 (6–8)	7 (6–8)	9 (8–10)	9 (8–9)	8 (7–9)	8 (7–8)
CO (l min^−1^)									
pREBOA_ETCO2_	6.1 (5.5–6.7)	2.8 (2.4–3.2)	3.0 (2.3–3.6)	3.3 (2.5–4.0)	3.2 (2.5–4.0)	4.8 (3.8–5.8)	6.0 (5.4–6.7)	5.2 (4.7–5.7)	4.6 (4.1–5.0)
pREBOA_SBP_	5.2 (4.5–5.6)	2.4 (2.2–2.6)	2.8 (2.5–3.1)	2.87 (2.5–3.2)	2.9 (2.5–3.3)	4.0 (3.4–4.5)	5.8 (5.2–6.5)	4.47 (3.8–5.1)	4.0 (3.2–4.5)

Hemodynamics during end-tidal carbon dioxide (ETCO_2_) targeted partial resuscitative endovascular balloon occlusion of the aorta (pREBOA, pREBOA_ETCO2_, *n* = 10) and proximal systolic blood pressure targeted pREBOA (pREBOA_SBP_, *n* = 10) in anesthetized, mechanically ventilated pigs. Data are means (95% confidence intervals)

HR: heart rate; MAP: mean arterial pressure; CVP: central venous pressure; CO: cardiac output

**Table 2:** Blood gases and blood samples during partial resuscitative endovascular balloon occlusion of the aorta

	Baseline	Hemorrhage	15 min REBOA	30 min REBOA	45 min REBOA	15 min reperfusion	60 min reperfusion	120 min reperfusion	180 min reperfusion
Arterial pO_2_ (kPa)									
pREBOA_ETCO2_	12.2 (11.7–12.6)	12.1 (11.5–12.7)	12.9 (11.9–14.0)	13.0 (11.8–14.1)	13.4 (12.2–14.5)	10.3 (9.7–10.8)	11.1 (10.5–11.6)	11.4 (10.7–12.1)	11.6 (11.0–12.3)
pREBOA_SBP_	11.8 (11.1–12.6)	12.1 (10.9–13.3)	13.5 (12.1–14.8)	14.1 (13.3–14.9)	13.7 (12.4–14.9)	10.8 (9.9–11.8)	10.8 (10.2–11.4)	11.3 (10.4–12.2)	11.6 (10.9–12.3)
Mes. ven. pO_2_ (kPa)									
pREBOA_ETCO2_	5.5 (5.0–5.9)	4.1 (3.8–4.4)^a^	4.4 (4.1–4.7)	4.2 (3.9–4.5)	4.6 (4.2–5.0)	6.1 (5.9–6.4)	6.4 (6.0–6.9)	6.5 (6.2–6.9)	5.8 (5.4–6.2)
pREBOA_SBP_	5.0 (4.5–5.5)	3.6 (3.3–3.9)^a^	4.2 (3.9–4.4)	4.3 (4.1–4.5)	4.4 (4.1–4.6)	6.5 (6.0–7.0)	6.9 (6.6–7.2)	6.1 (5.8–6.5)	5.7 (5.2–6.1)
VO_2_ (ml O_2_ min^−1^)									
pREBOA_ETCO2_	93 (73–113)	66 (48–85)	54 (43–65)	56 (45–67)	52 (41–63)	84 (68–102)	80 (64–96)	77 (61–93)	72 (56–89)
pREBOA_SBP_	87 (81–94)	56 (48–64)	52 (45–59)	53 (47–59)	51 (44–58)	87 (78–96)	88 (80–96)	80 (71–90)	75 (63–87)
VCO_2_ (ml CO_2_ min^−1^)									
pREBOA_ETCO2_	108 (98–117)	82 (72–91)	88 (82–93)	86 (79–94)	81 (74–88)	112 (104–119)	101 (94–107)	93 (88–98)	90 (83–96)
pREBOA_SBP_	102 (93–111)	77 (69–85)	78 (69–86)	79 (71–86)	72 (63–80)	112 (104–120)	104 (97–111)	95 (85–104)	87 (75–99)
DO_2_ (ml/min)									
pREBOA_ETCO2_	669 (577–762)	276 (222–331)	261 (186–335)	279 (195–363)	283 (192–373)	419 (276–562)	635 (517–753)	550 (479–622)	515 (438–590)
pREBOA_SBP_	560 (448–672)	216 (180–252)	239 (195–283	232 (182–282)	232 (184–281)	305 (240–370)	557 (430–683)	515 (392–637)	436 (266–605)
O_2_ER									
pREBOA_ETCO2_	38 (32–44)	76 (71–81)	64 (55–73)^a^	60 (51–68)	64 (52–76)	52 (45–59)	38 (33–43)	45 (31–59)	45 (35–54)
pREBOA_SBP_	38 (32–45)	80 (79–82)	52 (42–62)^a^	57 (48–67)	55 (44–66)	58 (51–66)	45 (39–50)	45 (41–50)	50 (39–61)
Creatinine (μmol L^−1^)									
pREBOA_ETCO2_	60 (55–64)						87 (76–97)^a^		93 (74–112)^a^
pREBOA_SBP_	62 (56–68)						99 (91–106)^a^		117 (105–130)^a^
Troponin (ηg L^−1^)									
pREBOA_ETCO2_	471 (290–766)						2641 (1166–5984)^a^		5366 (1768–5366)^a^
pREBOA_SBP_	513 (381–690)						7384 (3966–13,747)^a^		20,171 (8748–46,514)^a^
ALAT (μkat L^−1^)									
pREBOA_ETCO2_	1.1 (0.9–1.4)						1.1 (0.9–1.4)		1.2 (0.9–1.5)
pREBOA_SBP_	1.3 (1.1–1.5)						1.3 (1.1–1.5)		1.5 (1.3–1.7)
ASAT (μkat L^−1^)									
pREBOA_ETCO2_	1.0 (0.8–1.3)						1.8 (1.3–2.5)		3.0 (1.8–4.9)
pREBOA_SBP_	1.1 (0.9–1.4)						2.4 (2.1–2.8)		4.5 (3.1–6.4)
CK (μkat L^−1^)									
pREBOA_ETCO2_	19.3 (13.7–24.8)						22.3 (17.4–27.2)		28.3 (22.1–34.4)
pREBOA_SBP_	18.5 (14.9–22.1)						23.2 (18.9–27.6)		32.1 (26.2–38.0)

Arterial and mesenteric venous blood gases and arterial blood samples during end-tidal carbon dioxide (ETCO_2_) targeted partial resuscitative endovascular balloon occlusion of the aorta (pREBOA, pREBOA_ETCO2_, *n* = 10) and proximal systolic blood pressure targeted pREBOA (pREBOA_SBP_, *n* = 10) in anesthetized, mechanically ventilated pigs. Data are means (95% confidence intervals); ^a^denotes statistical difference between the groups

pO2: partial pressure of oxygen; Mes. ven. pO_2_: mesenteric venous pO_2_; VO_2_: oxygen consumption; VCO_2_: carbon dioxide production; DO_2_: oxygen delivery; O_2_ER: oxygen extraction ratio; ALAT: alanine aminotransferase; ASAT: aspartate aminotransferase; CK: creatine kinase

Oxygen consumption, O_2_ER and carbon dioxide production (VCO_2_) were similar in both groups (Table 2). Arterial pCO_2_ followed a similar pattern as ETCO_2_ (Fig. 3). The mesenteric pO_2_ differed significantly between the groups during hemorrhage, although with a numerically small difference (Table 2).

### Aortic occlusion

In the pREBOA_ETCO2_ group, ETCO_2_ was preserved on a statistically significantly higher level during the occlusion compared to the pREBOA_SBP_ group, whereas SBP was similar in both groups (Fig. 2). There were no statistically significant differences between the groups during occlusion in abdominal aortic blood flow, femoral arterial mean pressure, and metabolic markers; however, O_2_ER was slightly lower in the pREBOA_SBP_ group at 15 min of REBOA (Figs. 2, 3, Table 2).

The inflation volume of the balloon was adjusted from 2.3 (CI 1.8–2.9) ml at the start of occlusion to 2.8 (CI 2.1–3.4) ml at the end of occlusion in the pREBOA_ETCO2_ group, corresponding to 3.8 (CI 3.1–4.5) ml and 3.9 (CI 2.7–5.1) ml in the pREBOA_SBP_ group.

### Reperfusion

There was a statistically significant difference in arterial and mesenteric lactate concentrations between the groups during reperfusion, including at 1 h of reperfusion (primary outcome, Fig. 4). Arterial and mesenteric pH and potassium concentration showed a similar pattern, although only the arterial pH was statistically significant between the groups (Fig. 3). Oxygen extraction ratio was higher in pREBOA_SBP_ group during reperfusion (statistically significant at 120 min of reperfusion), in parallel to a slightly lower cardiac output in this group (not statistically significant, Tables 1 and 2).

Diuresis was absent at the end of REBOA in both groups and increased during reperfusion, with the maximal difference seen after 2 h, 55 (CI 26–118) µl kg^−1^ min^−1^ and 24 (CI 6–87) µl kg^−1^ min^−1^ in the pREBOA_ETCO2_ group and pREBOA_SBP_ group, respectively. The plasma creatinine and troponin concentrations were significantly lower in the pREBOA_ETCO2_ group after 1 and 3 h of reperfusion (Table 2). The plasma concentrations of CK and ASAT followed a similar pattern, although not statistically significant (Table 2).

The severity of ischemic injury in the small intestinal bowel segments differed between the subjects, ranging from no detectable damage to intact villi without epithelium and with crypt destruction. The dominating score was comparable between the groups with a median (25th–75th percentiles) of 3 (1–3.75) in the pREBOA_ETCO2_ group and 3.5 (1–4) in the pREBOA_SBP_ group.

## Discussion

This study compared the hemodynamic, metabolic and end-organ consequences of ETCO_2_ targeted and proximal SBP targeted pREBOA in a porcine model of hemorrhagic shock. The findings demonstrated that ETCO_2_ targeted pREBOA was as effective as proximal SBP targeted pREBOA in preserving hemodynamic stability but generated overall less metabolic insult; this included the primary outcome, arterial lactate concentrations at 1 h of reperfusion, and end-organ insults. These findings suggest a role of ETCO_2_ as a potential clinical tool when using pREBOA and may stimulate clinical studies on the subject, as well as the use of other metabolic and organ damage markers.

When using REBOA, achieving a balance between hemorrhagic control and ischemic burden is difficult in practice and there is, as yet, no consensus concerning how to use or titrate partial occlusion. Pressure targeted balloon occlusion has been the main focus in both preclinical and clinical settings; primarily SBP targeted, but also pressure gradient targeted occlusion [14, 19, 21, 25]. Distal blood pressure targeted occlusion has been studied by reducing the inflation volume from initial total occlusion to reach a pulsatile waveform or a target distal pressure, with a maximum of 10 mmHg being suggested [10, 19, 21, 34]. The pressure alone may, however, not represent the blood flow, since the resistance is unknown and may be dynamic [35]. In translational studies, a distal pressure increase of 10 mmHg has been suggested to correlate to a blood flow of 250–500 ml min^−1^, depending on shock state [10]. Recent studies have focused on distal blood flow instead of pressure and new balloons have been designed to facilitate titration to a targeted distal blood flow [23, 36–42]. A distal flow of 5–10% of naïve blood flow or 250–500 ml min^−1^ has been advocated sufficient to maintain viability of the viscera. However, the exact level of blood flow required to keep the distal organs perfused remains unclear. The association between distal ischemia and pREBOA has not been extensively studied. Matsumura et al. [43] demonstrated organ perfusion during pREBOA using an advanced computer tomography technique. The majority of these techniques are, however, difficult to use clinically or lack correlation to organ metabolism.

In a previous study by our group [31], aortic blood flow correlated well to ETCO_2_ and also to overall VO_2_ and VCO_2_ in a porcine study during normovolemia and during hemorrhage. As a result of hypoperfusion and maximum oxygen extraction, VO_2_ becomes oxygen delivery dependent and eventually drops below the critical oxygen delivery level. This is the point of onset of anaerobic metabolism and the rise in lactate levels [27, 44], and, therefore, the reason for choosing the arterial lactate concentration as a primary outcome. In this study, the lactate concentrations were significantly higher in the proximal SBP targeted group compared to the ETCO_2_ targeted group, indicating more anaerobic metabolism in the proximal SBP targeted group. Consequently, this group tended to be more acidotic, an aspect that affects the maintenance of vital organ function during shock. The targeted ETCO_2_ level used in this study was based on a prior study by our group in an attempt to maintain overall aerobic metabolism [31]. The targeted SBP level was based on clinically accepted permissive hypotension in trauma situations.

In this study, ETCO_2_ was preserved at a higher level in the ETCO_2_ targeted pREBOA group, while the SBP did not differ significantly during occlusion. The distribution of SBP in the ETCO_2_ targeted pREBOA group was notable and could be explained by the ETCO_2_ being used as the predetermined regulating factor. However, the SBP remained at a generally higher level in the ETCO_2_ targeted pREBOA group, even though it required a lower inflation volume. This could be the result of a gradual activation of the endogenous compensatory systems, such as the sympathetic nervous system and the renin–angiotensin–aldosterone system, in response to hypovolemia and occlusion of the distal organs [45]. The subjects responded diversely to hypovolemia; some responded with compensatory increased heart rate during the occlusion rather than during the hemorrhagic period, possibly due to individual responses to the anesthetic drugs, thus interfering with the balloon effect. The full mechanism of the physiological compensatory systems during pREBOA are not completely understood; however, higher angiotensin II levels during pREBOA compared to total REBOA have previously been suggested [46]. Higher systemic angiotensin II levels also concur with greater renal blood flow and glomerular filtration [46]. In clinical settings, pREBOA is currently most commonly performed as a stepwise degradation after initial total REBOA to establish clot stabilization [21, 47]. It might, however, be physiologically favorable to start with partial occlusion and gradually inflate, if necessary, to progressively stimulate the endogenous endocrine and sympathetic system to achieve less metabolic disturbance at acceptable central blood pressure.

The insignificant difference in aortic blood flow and the statistically significantly higher global O_2_ER, but lower mesenteric venous pCO_2,_ in the ETCO_2_ targeted group during aortic occlusion, may indicate greater blood flow to the lower body including the intestines. Higher diuresis and significantly lower creatinine levels were also found during reperfusion in this group. This is in accordance with previous studies comparing total REBOA and permissive perfusion [48]. Likewise, the troponin levels were significantly higher in the SBP targeted group, further suggesting greater tissue damage. These results may indicate that pREBOA targeted by proximal SBP alone requires more extensive occlusion than is necessary at the expense of the perfusion of distal organs.

### Limitations

This study has some limitations. No a priori statistical power analysis was performed, although an interim analysis was carried out to calculate the number of animals required. Another limitation is related to the implementation being performed on healthy pigs in a single trauma setting without ongoing bleeding. In a clinical setting, ETCO_2_ may be influenced by underlying pulmonary disease, thoracic trauma or ongoing trauma. When constant ventilation cannot be maintained, ETCO_2_ is difficult to interpret due to confounding factors. However, it may be possible to calculate an estimate of VCO_2_ using minute ventilation and ETCO_2_ concentration to make the interpretation independent of minute ventilation, thus allowing non-constant ventilation. In addition, this study investigated the short-term but not the long-term effects of pREBOA using the different techniques. Further studies are needed to evaluate the long-term effects on organ failure.

This study does not encourage ETCO_2_ as a single measurement tool for pREBOA in the emergency room or prehospitally, but as a supplementary tool to other hemodynamic measurements to estimate the metabolic sequelae caused by aortic occlusion. This is, to our knowledge, the first study using a metabolic variable to target pREBOA and further studies are needed to evaluate the clinical value of ETCO_2_ during pREBOA.

## Conclusion

In a porcine model of hemorrhagic shock, ETCO_2_ targeted pREBOA caused less metabolic disturbance and end-organ damage compared to proximal SBP targeted pREBOA, with no disadvantageous hemodynamic impact. End-tidal CO_2_ should be investigated in clinical trials as a complementary clinical tool to mitigate ischemic–reperfusion injury when using pREBOA.

## Data Availability

The data sets used and analyzed during the current study are available from the corresponding author on reasonable request.

## Abbreviations

REBOA: Resuscitative Endovascular Balloon Occlusion of the Aorta
pREBOA: Partial REBOA
ETCO_2_: End-tidal carbon dioxide
SBP: Systolic blood pressure
pREBOA_ETCO2_: ETCO_2_ targeted pREBOA
pREBOA_SBP_: Proximal SBP targeted pREBOA
EVTM: Endovascular Resuscitation and Trauma Management
VO_2_: Oxygen consumption
DO_2_: Oxygen delivery
O_2_ER: Oxygen extraction rate
MV: Minute Volume
pCO_2_: Partial pressure of CO_2_
VCO_2_: Carbon dioxide consumption
pO_2_: Partial pressure of O_2_
